# Sensor-Assisted Weighted Average Ensemble Model for Detecting Major Depressive Disorder

**DOI:** 10.3390/s19224822

**Published:** 2019-11-06

**Authors:** Nivedhitha Mahendran, Durai Raj Vincent, Kathiravan Srinivasan, Chuan-Yu Chang, Akhil Garg, Liang Gao, Daniel Gutiérrez Reina

**Affiliations:** 1 School of Information Technology and Engineering, Vellore Institute of Technology, Vellore 632014, India; nivedhitha.m2019@vitstudent.ac.in (N.M.); pmvincent@vit.ac.in (D.R.V.); kathiravan.srinivasan@vit.ac.in (K.S.); 2 Department of Computer Science and Information Engineering, National Yunlin University of Science and Technology, Yunlin 64002, Taiwan; 3 State Key Lab of Digital Manufacturing Equipment & Technology, School of Mechanical Science and Engineering, Huazhong University of Science and Technology, Wuhan 430074, Chinagaoliang@mail.hust.edu.cn (L.G.); 4 Electronic Engineering Department, University of Seville, 41092 Seville, Spain; dgutierrezreina@us.es

**Keywords:** correlation-based feature selection, random forest, weighted average ensemble, major depressive disorder, smartwatch sensor

## Abstract

The present methods of diagnosing depression are entirely dependent on self-report ratings or clinical interviews. Those traditional methods are subjective, where the individual may or may not be answering genuinely to questions. In this paper, the data has been collected using self-report ratings and also using electronic smartwatches. This study aims to develop a weighted average ensemble machine learning model to predict major depressive disorder (MDD) with superior accuracy. The data has been pre-processed and the essential features have been selected using a correlation-based feature selection method. With the selected features, machine learning approaches such as Logistic Regression, Random Forest, and the proposed Weighted Average Ensemble Model are applied. Further, for assessing the performance of the proposed model, the Area under the Receiver Optimization Characteristic Curves has been used. The results demonstrate that the proposed Weighted Average Ensemble model performs with better accuracy than the Logistic Regression and the Random Forest approaches.

## 1. Introduction

As we live in modern times where computer jobs and fast food chains are used to enhance life, no one is ready to consider exercising or taking time out [1]. With all the stress happening, it is more than typical that the stress comes with anxiety and depression. People these days are confused with what they are doing and do not have a proper vision, which makes them puzzled. In the long term, this can end up in depression [2]. The more dangerous part is that the person may not know they are experiencing depression [3]. The prevailing thought among people is that they should take care of their physical health, but the fact is mental health is just as important as physical health, and in fact, it is more important as anything that affects mental health will eventually affect the physical, too [4]. The reason for Clinical Depression or Major Depressive Disorder (MDD) is still unknown, but experts say it can be one among the following: substance abuse, tragic stressful events, family history, present health issues, or medications [5]. The symptoms usually include long term sadness, hopelessness, thoughts of suicide, and other psychotic symptoms [6]. MDD should be handled at an early stage because there are chances it could lead to suicide or result in mania or bipolar disorder (also known as manic depression), which is more dangerous [7].

Nowadays, mental health issues seem to be growing day by day [8]. It would be easier to treat if there are methods to anticipate the mental health issues [9]. There are two complications in treating a depressed individual. First, it is difficult for the person to identify that MDD is a perilous phenomenon and fail to realize that their mental health is degrading day after day. Then even if they realize the fact that they are affected by depression, getting them to professional help is difficult due to lack of motivation, cost, and mobility [10]. If the patients do seek professional help, the psychiatrists find it difficult to diagnose depression, as it is comorbid, i.e., it occurs with other disorders. Also, the initial level of treatment is a questionnaire, which can be subjective. The psychiatrists may not know whether the patient is truthful or not [11].

Digital smartwatches are a powerful tool in analyzing the behavior of the wearer and use the data for various studies. For instance, how long an average person works out in a day, average calories burnt per day, and much more [12]. Nowadays, researchers are focusing on using smartwatch data to analyze the mental health of individuals. When it is about mental health and seeking help, almost 40% percent of the population reported to have experienced depression or anxiety in their life but never looked for help, and the reason, perhaps, is they did not know where to begin with [13]. That is where the smartwatches and apps come into the picture, as they can gather data without any effort from the wearer. Also, this overcomes the issue with the questionnaire, which can result in a subjective outcome [14]. Smartwatches help in real-time monitoring, and high-frequency data offers an objective and a different variation from the traditional subjective self-reported data.

Machine Learning is one of the tools of Artificial Intelligence [15,16]. Artificial Intelligence is a simulation done with the help of machines, mainly computers of human intelligence. This simulation includes constant learning, reasoning, and adapting by self-correction. Machine Learning is derived from AI, which gives the machines the ability to teach themselves without anyone instructing it what to do [11]. Machine learning algorithms learn themselves from the given data and apply the gained knowledge to make predictions. For diagnosing depression at an early stage, it is better to focus on the overall pattern in the data rather than looking at individual attributes [17]. Machine Learning has algorithms that are experts in finding the pattern from the data. Machine Learning techniques are very good at discovering the best combination of features from the data [5].

The Machine Learning models based on the way they handle the data are classified into three major categories: Supervised Learning, Unsupervised Learning, and Reinforcement Learning [18,19]. The process of prediction or classification involves four stages: collecting data, pre-processing it to handle the missing values and reduce the noise, select the essential features, then implement a model suitable for the data in hand [20].

In this study, we have done all three stages and designed a machine learning model for diagnosing Clinical Depression or Major Depressive Disorder at an early stage where it can be treated easily. We have used two types of data, including objective and subjective. The objective data is recorded from the smartwatch sensors, and the subjective data is taken from the participants through self-rated questionnaires. In the age of modern technologies ruling, we have made use of electronic smartwatches, which has an accelerometer component for gathering data about the participants [21].

Once the features are decided, we have proposed a Weighted Average Ensemble machine learning model by combining the Logistic Regression and Random Forest method. It is always better to combine models than to implement it as individuals, as one’s limits would be compromised by the other. Here, we have proposed a Weight Average Ensemble model by combining the Logistic Regression and Random Forest Model to improve the prediction accuracy.

The key contributions of this work are summarized as follows:

(i) To the best of our knowledge, a Weighted Average Ensemble machine learning model is developed for the first time in this paper detecting Major Depressive Disorder (MDD) using an integrated feature set, and its performance is justified through experimental results.
(ii) A unique integrated feature set is formulated by combining the features from the questionnaire, and the smartwatch sensor encompassing a heart rate monitor.
(iii) The gathered data is pre-processed to handle the missing values with the help of Mean Imputation, and then the significant features are selected using the Correlation-based Feature Selection technique.
(iv) The proposed Weighted Average Ensemble model surpasses the logistic regression, and the random forest approaches in terms of the area under the receiver operating characteristic (ROC) curves measure.
(v) It can be observed from the experimental results that the Weighted Average Ensemble model performs better in terms of accuracy, precision, recall, specificity, and FMeasure in due comparison with Logistic regression and Random Forest Models. Furthermore, the proposed model also illustrates a superior performance with an accuracy of 99.01%.

The remaining portion of the paper is structured as a Review of Literature, Dataset Description, Methodology, Results and Discussion, and Conclusion.

## 2. Review of Literature

It is an arduous task to differentiate between Parkinson’s disease’s postural re-emergent tremors from essential tremors. So, the authors in this study [22] have used a smartwatch to monitor and record the tremors. They have considered 41 patients for the study, and the recordings were done for an accelerometer from a smartwatch as well as an analog accelerometer in parallel. With the results they got, they concluded that the smartwatch shows more prominence than the analog device. It provides diagnostically accurate and relevant information on postural tremor when compared with other analog methods.

In newborn babies, it is crucial to find out the Initial Heart Rate [23,24]. The heart rate detection using auscultation of heart and palpation of umbilical cords were found to be inaccurate and unreliable [25]. Also, late, the NRP (Neonatal Resuscitation Program) has recommended using pulse oximetry, but that took a long time to detect the heart rate, and sometimes it is even a failure. Then they came up with ECG, which is faster but expensive. Hence, to overcome these issues, the authors have adopted a smartwatch technique to find out the heart rate of newborn babies, which is accurate and inexpensive.

In this approach [14], the authors have implemented a machine learning-based approach using the smartwatches for activity recognition. Furthermore, this involves matching activities with a times series sensor from a smartphone or smartwatch. The activities include Walking, Sitting, Jogging, Standing, Climbing, Eating, and many other activities that involve hands and does not involve hands. They have used the WEKA tool for implementing the machine learning models. The algorithms employed are Random Forest, IB3 instance-based, J48 Decision Tree, Multi-Layered Perceptron, and Naïve Bayes. They have compared the performance of the accelerometer and gyroscope of the smartwatch and found out that the accelerometer of the smartwatch performs better than the gyroscope.

For people with mental disorders such as dementia, it is essential to monitor patients to ensure whether they are performing the proper exercise and the necessary amount of sunlight. For their health and safety, it is imperative to monitor them continuously. The authors in this study [26] have developed a smartwatch with features such as accelerometer, GPS, and illumination sensor. Moreover, this will help in monitoring patients with dementia. The proposed algorithm effectively identifies the amount of exercise the patients are taking and everything else that is required, and it shows 96% of success with its experimental results.

In this approach [27], the authors have implemented a machine learning model by gathering sensor data from the electronic smartwatches to predict the alcohol content in blood in real-time. They have also developed an Android application to connect to the sensors and also to store data from the sensor. They have implemented both classification and regression models. For the regression problem, they have used Linear Regression and Artificial Neural Networks, and for the classification problem, they have using Support Vector Machine and Logistic Regression. They have evaluated their model using RMSE for regression and recall, FMeasure, and Precision for classification. In the end, they found that this problem is tackled effectively if it is considered as a classification problem, and among the two classification algorithms, SVM performed better.

It is vital to diagnose depression at an early stage in order to treat it. In this approach, the authors [28] have done a non-linear analysis of EEG signals to differentiate depressed patients from healthy individuals. They have considered 45 depressed patients and extracted features from the EEG signals. They have used three machine learning classifiers, such as K-Nearest Neighbour, Linear Discriminant Analysis, and Logistic Regression. The results show that the highest classification accuracy is achieved by the Logistic Regression classifier (90%).

The studies mentioned above are sensor-based and machine learning related approaches applied to various healthcare domains. There are very fewer works on sensor-based approaches along with machine learning applied in classifying or predicting MDD. The major drawback of using the smartwatch sensors is that various factors would affect the sensors and heart rate-monitors of the wearer. The traditional method to diagnose depression is using a questionnaire. However, most of the time, the questionnaire seems to be subjective, whereas the sensors generate objective data, i.e., it requires no effort from the subject. Hence, in this study, we have used the combination of both (sensors and questionnaire) to collect the subjective and objective data and validate the results in classifying the subjects with MDD.

## 3. Methodology

In this study, we have used the Mi band-3 smartwatch to detect the behavior and heart rate of the participants in diagnosing MDD. The participants were given a questionnaire to fill every day in the morning for one week. The participants were made to wear the smartwatch all the time for one week including the weekend, even when they are sleeping. The smartwatches can measure the sleep patterns and have the component for continuous heart-rate monitoring. The participants in this study are a combination of male and female, with an average age of 40, weight of 60 kg, and height 176 cm.

**Figure 1:**
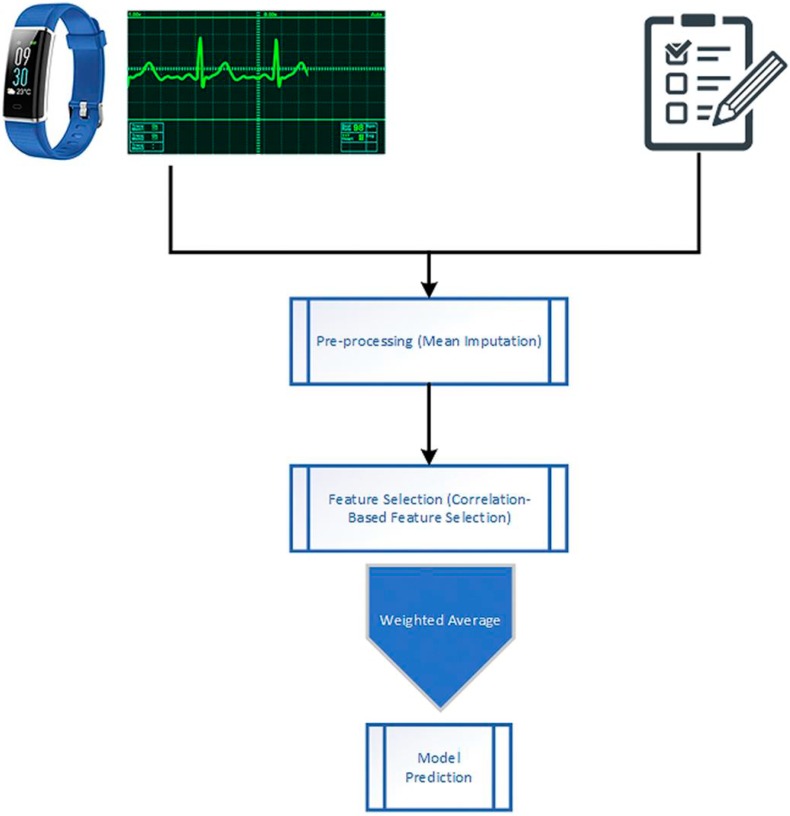
Architectural Diagram of the Proposed Model.

The methodology comprises of three phases: Pre-processing of data, selecting essential features from the data, and applying Machine Learning models individually on the high discriminative features, and also applying the proposed weighted average ensemble machine learning model on the data, then comparing it with the individual implementation of the base models. In this section, we explain the process by which we did the classification. Figure 1 illustrates the overall architecture of the study.

### 3.1. Dataset Description

We have collected data from 500 average aged people who complained about mood swings. We used data from the Questionnaire, Smartwatch, and Heart rate monitor. The data collection involved 500 people, and among the 500 records, we rejected 50 because of technical issues. Together the dataset consists of 35 features, which are later reduced using feature selection techniques. We have used the Hamilton Depression Rating Scale [29] for the questionnaire and the features from the accelerometer of the smartwatch. All these features are combined after feature selection and then passed through machine learning models.

### 3.2. Hamilton Depression Rating Scale

Hamilton Depression Rating Scale (HDRS) is the most widely used self-rating report for assessing depression. The questionnaire consists of 21 items to rate the severity of depression. Among the 21 items, 17 are severity measures for the depression while the remaining four are symptoms related to depression, but not considered as severity measures, for instance, paranoia or obsessive symptoms. The interpretation of the scores in HDRS is 0–7 is normal, 8–16 is mild depression, 17–23 is moderate depression, and greater than 24 is considered as severe depression [30].

### 3.3. Smart Watch Sensors

A smartwatch is similar to the wristwatch in appearance, but does so many things other than keep time. The digital smartwatches, Bluetooth is enabled, and the features can be extended to smartphones. In such cases, the users can use the smartwatch for reading the messages, answer phone calls, check the weather, and many advanced features [12]. In addition to these benefits, the smartwatches help in analyzing the behavior of the wearer and determine their mental health. The smartwatch has two sensors, Accelerometer and Gyroscope [13] for recording the data from the gestures that the wearer makes.

#### 3.3.1. Accelerometer

The accelerometer is used in the smartwatches to detect the movement and orientation of the wearer. With accelerometer in the smartwatch, around two dozens of gestures and actions are detected, irrespective of one or two hands [20]. These gestures are later mapped to the controls or software applications. The typical variation used in the smartwatch is the Tri-axial, which keeps track of the physical activities of the wearers. Unlike the uniaxial variation, which records only the up and down movements, the Tri-axials record up and down, side-to-side, and back-and-forth movements [18].

#### 3.3.2. Gyroscope

Gyroscope sensors are also used to measure the orientation and the angular velocity of the wearer. Gyroscope sensors have advanced functionalities than the accelerometers. These sensors can track the lateral and also the tilt orientations. On the other hand, accelerometers can track only the linear motions [31]. The design of the gyroscope has a rotating disk called the motor, which is mounted on a spinning axis. This sensor determines the orientation of the wearer with the help of Earth’s gravitational force [32].

#### 3.3.3. Heart-Rate Monitor

The heart-rate monitors used in the smartwatches are designed based on a principle called Photo Plethysmography (PPG) [32]. PPG is the process of sending a shining light through the skin and measuring the scattered amount in the blood. The scattering is based on the blood flow dynamics such as the blood volume or the pulse rate. The three essential components in measuring the heart-rate are Optical Emitter (LEDs to send the light), Digital Signal Processor (captures the refracted light from the user and converts them into heart rate data), and Accelerometer (used in combination with the DSP in measuring the motions). With the help of DSP and Accelerometer data, the calories burned, blood pressure, and oxygen levels in the blood can also be measured [12].

### 3.4. Pre-Processing

Data pre-processing is a technique to remove the missing values and irrelevant values from the data. Data collected from various sources will usually have missing values, redundant and irrelevant data, which will reduce the prediction accuracy of the model [33]. It is essential to clean the data before applying any algorithm to improve the performance of the model [34].

In this study, we have used a single imputation method, which is mean imputation. In mean imputations, the missing values in certain features will be replaced by taking the mean of the available values in the feature column [35]. This is a simple yet effective method for handling missing values.

### 3.5. Feature Selection

Feature selection is also a part of pre-processing, where the number of features in the original dataset is reduced [36]. The reason for performing feature selection is to avoid features that do not affect the target variable [37]. Feature selection is a critical phase in any machine learning process, which significantly impacts the performance of the model. Partially irrelevant or irrelevant features are considered to negatively impact the model accuracy [38]. It is the process of selecting (automatically or manually) the critical feature that contributes more to the target variable or prediction variable [39]. If the model learns from the data that has missing values and irrelevant values, then the model would become weak.

In this study, we have used a correlation-based feature selection method to remove the features that interact the most and not having any part in the classification or prediction process. The correlation-based feature selection technique will identify the best possible subset from the whole feature set that might have a potential impact on the model outcome. The features are eliminated based on their inter-feature correlation threshold. This technique takes into consideration the individual attribute abilities and the redundancy among them with the help of merits score of subsets [40].

### 3.6. Machine Learning Models

Machine learning is one of the applications of Artificial Intelligence, which makes the system capable of making its own decision by learning from past events, and also improve and adapt to future changes [8]. Based on the way of handling the data, machine learning mainly consists of three types. Primarily, Supervised Learning where the data is labeled, and the output is known already. Secondly, Unsupervised Learning where the data is unlabelled, and the output is decided later from the inferences. Finally, Reinforcement Learning, which is based on the feedback mechanism, the algorithm is in such a way that it interacts with the environment, finds the rewards or errors [8,20].

**Figure 2:**
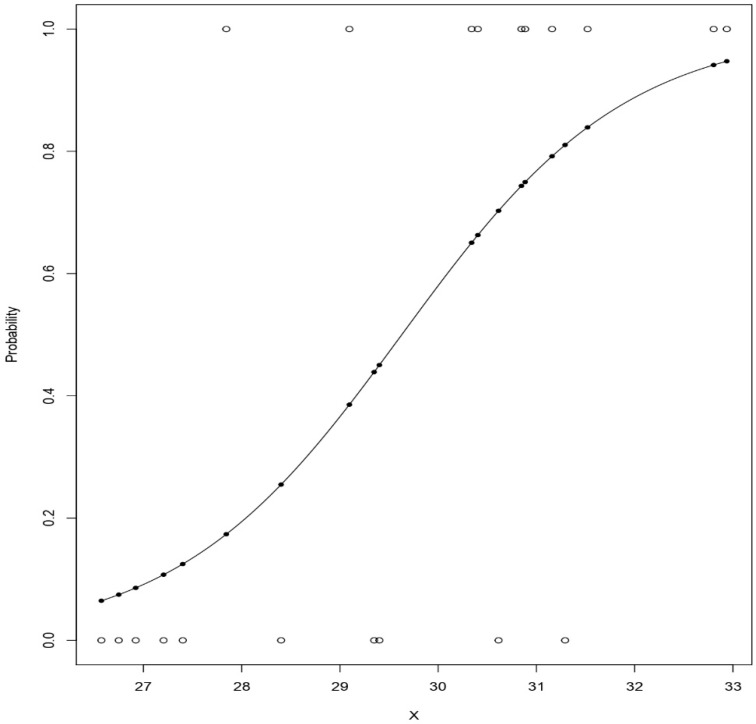
Sigmoidal Curve. Description: Here, ‘x’ can be any dependent attribute.

In this study, we have implemented two supervised machine learning methods: Logistic Regression and Random Forest, along with the proposed weighted average ensemble model. Logistic Regression is a statistical method usually used in cases where the target variable is categorical (i.e., dichotomous, 1 or 0) [41]. It is used widely for predictive analysis also to interpret the relationship between one binary variable that is dependent and other independent or nominal variables. The logistic regression is represented by a sigmoidal curve [42]. Figure 2 shows a sample sigmoidal curve where ‘x’ can be any dependent attribute; if the curve goes towards the positive side, then the prediction becomes 1. If it goes towards negative, then the prediction becomes 0. The logistic regression equation is given by [43],

(1) $$P\left(A\right)=\frac{1}{1+e^{-\left(a_{0}+a_{1}B\right)}}$$

where,

*P*(*A*)—Is the probability of A (Dependent Variable)
a_0_—moves the curve right and left
a_1_—Slope
*B*—Nominal Variable or Independent Variable.

**Figure 3:**
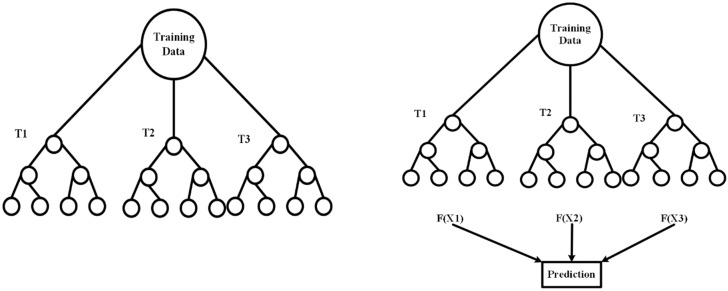
The schematic diagram for Random Forest Approach.

Random forest is the most widely used machine learning model that can be used for both regression and classification. Random forest is an ensemble of several decision trees, and the prediction is made as a result of taking an average of all the predictions from the decision trees [44]. The random forest has a root node which separates the sample classes, which is further divided into several branches [45]. The classifications are done using the training data, and the predictions are accomplished using the testing data. Figure 3 represents a sample schematic of the random forest approach.

Also, we have implemented the weighted average method by combing the Logistic Regression model and the Random Forest model to improve the prediction accuracy. Two is always better than one. A weighted average is considered to be an extension to the average method where multiple predictions are made on the data points, and then an average of all the predictions would be taken as the final prediction value. On the other hand, in the weighted average, each data point would be given a pre-defined weight in order to show their importance in the prediction, and then the weighted average of all the data points is considered to be the final prediction. In a weighted ensemble, each member of the ensemble contributes to the final prediction. In the case of class label prediction, the mode of the predictions by the members is used for final prediction. In the case of class probability prediction, argmax of the summed probabilities of every class label is used for the final prediction.

## 4. Results and Discussion

The dataset consists of 500 records combining the questionnaire and smartwatch encompassing the heart monitor sensor data. Of the 500 data, we have rejected 50 due to technical errors and used 450 records. The 450 records are pre-processed using mean imputation technique to handle all the missing values in the accelerometer data from smartwatch data as well as the questionnaire data. After correcting the missing values, the feature selection technique is employed. The feature selection technique is applied to data from the questionnaire and also the accelerometer data from a smartwatch. The data is segregated into a training set and testing set where the training is done with the training set, and the predictions are made using the testing set. The composition of training and testing data is 80% and 20% with 10-Fold cross-validation.

The selected features from the questionnaire excluding the Outcome feature, which is the Target variable, is:

1. Feeling Sad
2. Feeling Irritable
3. Feeling Anxious about Tense
4. Response to Mood to Good or Desired Events
5. The mood in Relation to the Time of Day
6. Thoughts of Death or Suicide
7. Capacity for Pleasure or Enjoyment
8. Bodily Symptoms
9. Panic/Phobic Symptoms

The selected features from the accelerometer of the smartwatch are:

1. Standard Deviation
2. Root Mean Square
3. Root Sum Square
4. Upper Quartile
5. Lower Quartile
6. Kurtosis

Along with the nine features from the questionnaire and six from the accelerometer heart rate from the heart rate monitor, there are totally 16 features selected by the correlation-based feature selection technique.

After removing the missing values and selecting the critical features, two machine learning techniques are implemented, Logistic Regression and Random Forest. The classifiers are evaluated using Confusion Matrix, Accuracy, and cut-off points along with the AUC-ROC Curve (Area under the Receiver Optimization Characteristics). Confusion Matrix is a table used for evaluating a model whose truth-values are known. The confusion matrix consists of features such as Accuracy, Specificity, Sensitivity, Precision, and Recall. The formula and definition of confusion matrix features are given in Table 1. We have utilized the AUC-ROC curve, too, which is the most critical metric for evaluating the performance of the classification model. In AUC-ROC, AUC is the measure or degree of separability and ROC is the probability curve. When the model generates higher AUC, it implies that the model is efficiently classifying the patients without disease and with disease. The AUC-ROC curve is plotted with the True Positive Rate (TPR) on the y-axis against the False Positive Rate (FPR) on the x-axis. AUC-ROC says the capability of the model in differentiating the classes.

**Figure 4:**
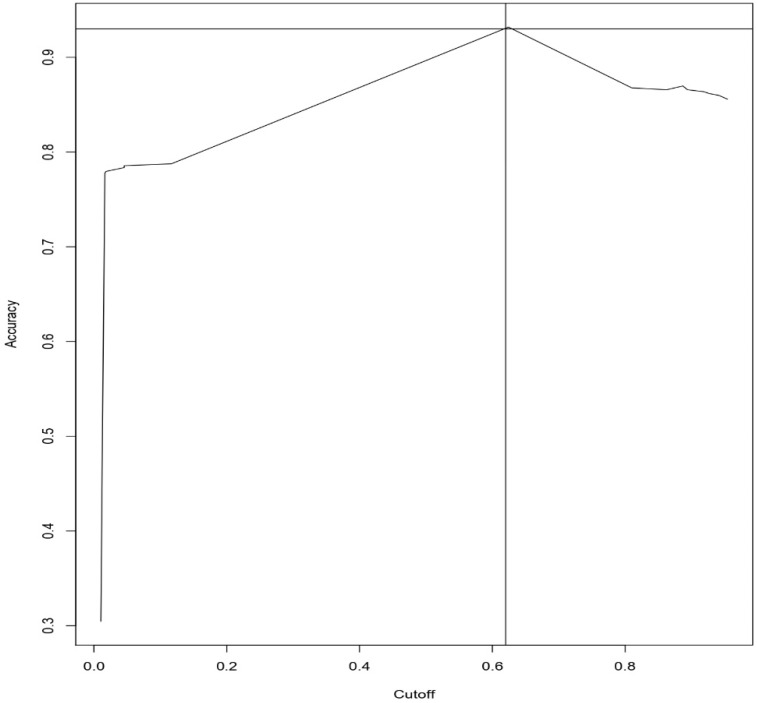
Accuracy Vs. cut-off curve for Logistic Regression Approach.

**Figure 5:**
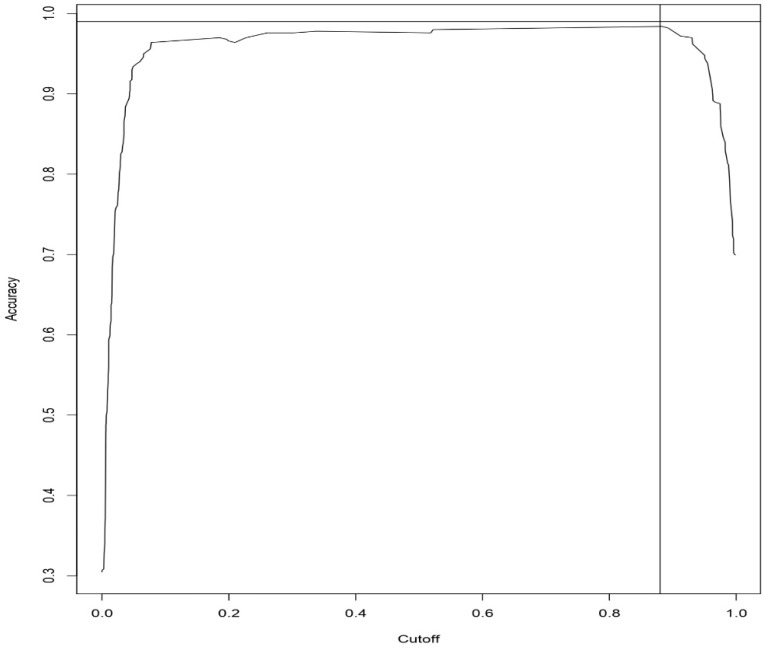
Accuracy Vs. cut-off curve for Random Forest Approach.

Figure 4 and Figure 5 represents the Accuracy vs. cut-off plot for Logistic regression and Random Forest, respectively. With the results, we have found that the Accuracy and cut-off for Logistic regression are 93% and 62%, respectively. The Accuracy and cut-off for Random Forest are 98% and 88%, respectively.

**Figure 6:**
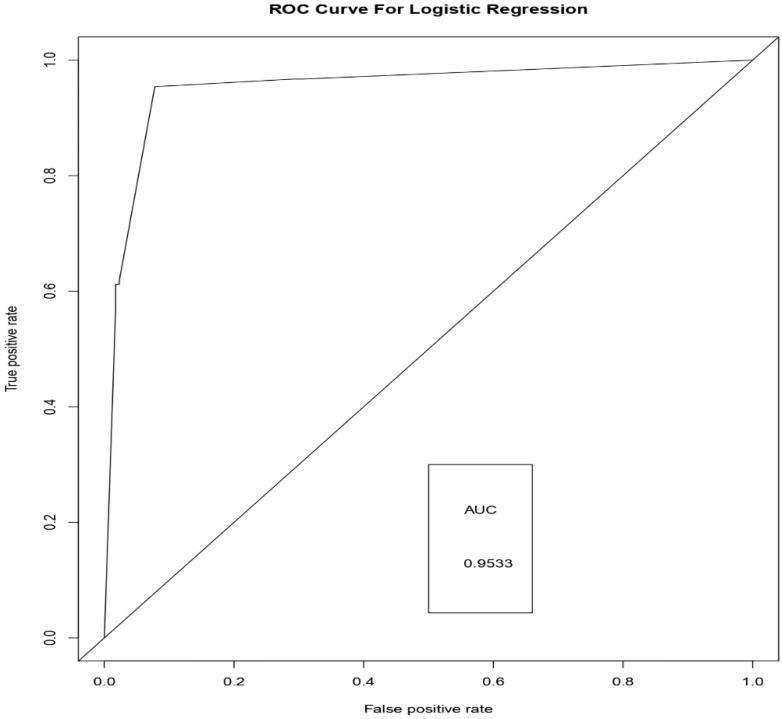
AUC-ROC curve for Logistic Regression Approach.

**Figure 7:**
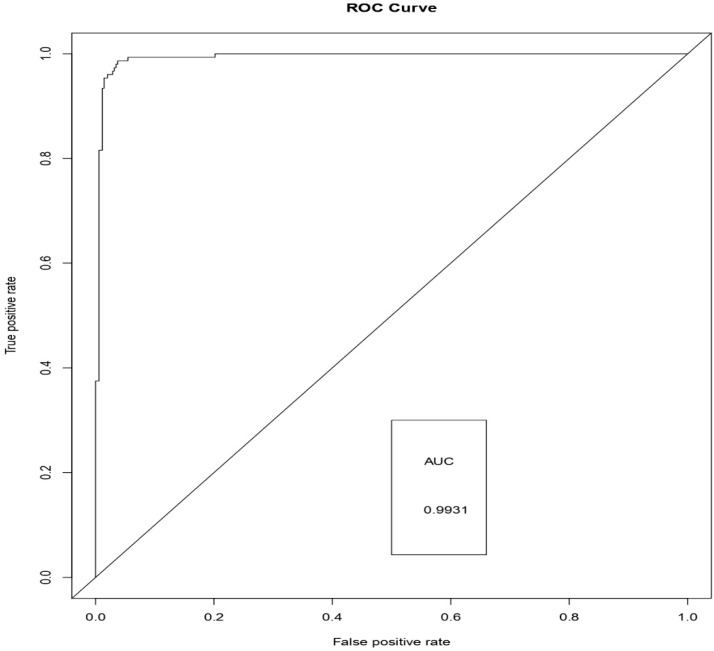
AUC-ROC for Random Forest Approach.

**Figure 8:**
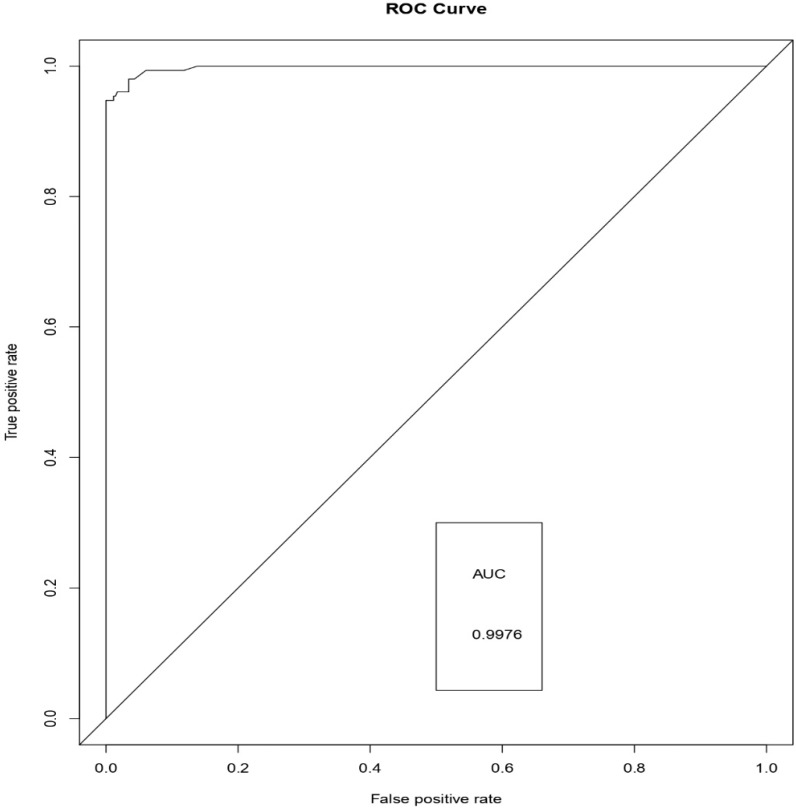
AUC-ROC curve for Weighted Average Ensemble Model.

**Figure 9:**
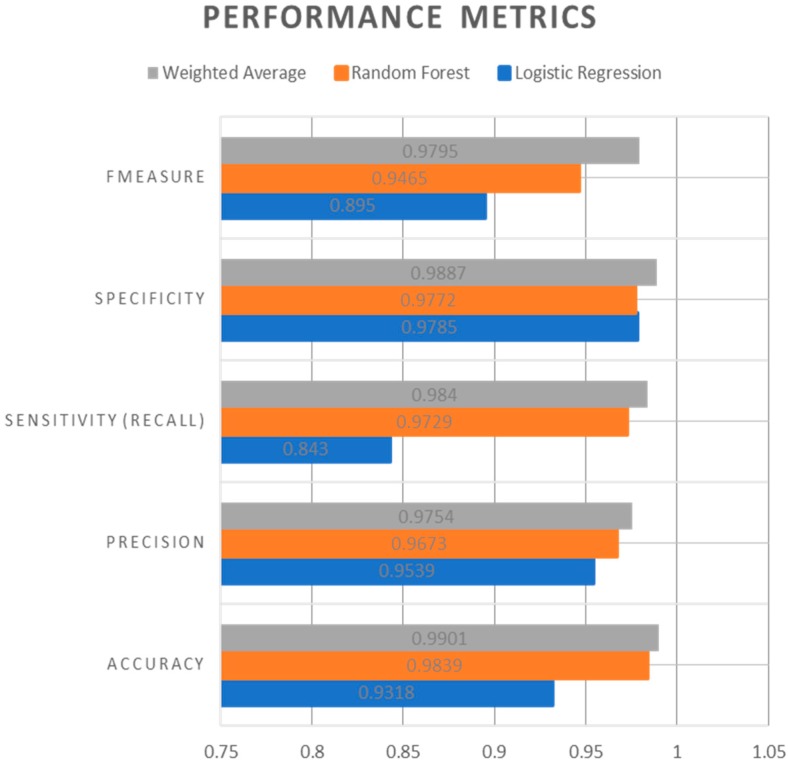
Performance comparison between Logistic Regression Model, Random Forest Approach, and the proposed Weighted Average Ensemble Model.

Figure 6, Figure 7 and Figure 8 represents the AUC-ROC curve for Logistic Regression, Random Forest approaches, and Weighted Average Ensemble Model, respectively. The Area under the curve for Logistic Regression is found to be 95%, for Random Forest Approach, it is 99.31%, and for the Weighted Average Ensemble Model, it is 99.76%, which is a reasonable improvement. From Figure 9, we observe that the Weighted Average Ensemble model shows better results when compared to Logistic Regression and Random Forest Approaches.

The values of the confusion matrix for Logistic Regression, Random Forest, and Weighted Average Ensemble Models are given in Table 2. From this table, we can realize that the performance of the Random Forest machine learning model is more accurate and useful than the Logistic Regression model. Random Forest Approach is 98% accurate, and Logistic Regression Model is considerably less, which is 93% accuracy. We can also notice that the Weighted Average Ensemble model performs marginally better than the two models implemented individually. We found out that, in the Weighted Average Ensemble model, as the error rates are minimized, the algorithm provides faster results than the individual implementations of Logistic Regression and Random Forest Approaches.

## 5. Conclusions and Future Work

In this study, we have implemented the machine learning models to diagnose Clinical Depression at the earliest possible time. We have gathered data from the electronic smartwatch and questionnaire. The smartwatch has a sensor called Accelerometer. We have used accelerometer sensor data and combined it with the features from the questionnaire. We have used the Hamilton Depression Rating Scale as a questionnaire, but it is subjective data, hence why we used the smartwatch sensor data, which was attached to the wrists of patients. The data collected are pre-processed using the mean-imputation technique, and then correlation-based feature selection technique is applied separately on questionnaire data, and accelerometer data and then the selected features are combined together, and then Logistic Regression and Random Forest machine learning models are applied on the features and then combined the two models to form a Weighted Average ensemble model. For evaluating the implemented model, we have employed Confusion Matrix and Area under the Receiver Optimization Characteristics, and the results show that the Random Forest model performs better in predicting the depressed patients than the Logistic Regression model and the Weighted Average Ensemble performs better than the two models.

In this implementation, we have considered only the accelerometer sensor of the smartwatch. The accelerometer will help in orientation only when the object is relative to the earth’s surface. For instance, when the object is under free fall, accelerometer data will show zero acceleration. The other sensor which is used in the smartwatches is the Gyroscope, which will sense rotation and movements that are not relative to the earth’s surface. In our future works, we will consider both the sensors of the smartwatch for better accuracy and employ various other machine learning models to explore this area of research further.

## Figures and Tables

**Table 1:** Confusion Matrix Components.

Confusion Matrix	Definition	Formula
Accuracy	It is the ratio of correctly classified to the whole set. For instance, which answers the question: How many patients did we correctly diagnosed as depressed out of all the patients?	TN + TP/All
Precision	It is the ratio of correctly classified positive subjects to all the positive subjects. For instance, which answers the question: How many of the patients whom we named as depressed are actually depressed?	TP/TP + FP
Sensitivity (Recall)	It is the ratio of correctly classified positive subjects to all those who have the disease in reality. Which answers the question: Of all the depressed people in the dataset, how many did we correctly predict as depressed?	TP/TP + FN
Specificity	It is the ratio of correctly classified negative subjects to all the healthy subjects in reality. Which answers the question: Of all the healthy people in the dataset, how many we correctly predict as not depressed?	TN/TN + FP
FMeasure	It is a combination of both recall and precision. Harmonic average.	2 × (Precision × Recall)/(Recall + Precision)

**Table 2:** Performance Evaluation of LR, RF, and the proposed Weighted Average Ensemble Model.

Performance Metrics	Logistic Regression	Random Forest	Weighted Average
Accuracy	0.9318	0.9839	0.9901
Precision	0.9539	0.9673	0.9754
Sensitivity (Recall)	0.8430	0.9729	0.9840
Specificity	0.9785	0.9772	0.9887
FMeasure	0.8950	0.9465	0.9795
